# Scaling up the global Green Bond market: Integrating global policy frameworks with stakeholder perceptions from Australia and the United Kingdom

**DOI:** 10.1371/journal.pone.0338998

**Published:** 2026-10-05

**Authors:** Madurika Nanayakkara, Sisira Colombage

**Affiliations:** 1 Department of Commerce and Financial Management, Faculty of Commerce and Management Studies, University of Kelaniya, Dalugama, Sri Lanka; 2 Institute of Innovation, Science, and Sustainability, Federation University Australia, Berwick Campus, Berwick, Australia; Yamanashi Gakuin University: Yamanashi Gakuin Daigaku, JAPAN

## Abstract

The Green Bond (GB) market has not been as effective as anticipated in tackling the global climate change crisis. There is a substantive investment gap worldwide in meeting the financing needs of green projects. Thus, this study reviewed key publications of international market players from 2007 to 2021 to identify how key stakeholders perceive the GB market. We employed a qualitative research approach using document analysis. In addition to this, a number of interviews were conducted to validate the findings by providing suggestive and supportive evidence. The findings indicate structural constraints affecting both supply and demand dynamics in the GB market. This study contributes to the literature by integrating documentary evidence and stakeholder perspectives into a unified conceptual framework that explains the institutional and market barriers affecting the global scaling of Green Bond markets and identifies implications for future policy development.

## 1. Introduction

There has been a call for urgent action regarding climate change issues due to its threat to sustainable development worldwide [1,2]. Many renewable energy developers of wind and solar projects prefer to finance their work by issuing Green Bonds (GBs) rather than generating funds from traditional capital sources, such as equity or conventional debt. However, most of these projects are performing differently than expected due to the need for more financing. Despite the number of eco-friendly investments evolving worldwide, the climate challenge continues to progress, and the benefits of sustainable investments need to be more convincing in the market. Although GBs have appeared as a critical solution to persuade both energy producers and investors about the benefits of eco-friendly investments, the overly complicated GB frameworks and many other unidentified matters have hampered the development of the GB market [3,4]. Thus, linking renewable energy and the GB market would provide promising prospects for investors, developers, and sustainable development. However, the concept of GB needs to be appropriately defined and presented to market participants for the success of these prospects. Thus, this study aims to recognize the ways that could be used to scale up the GB market by identifying the stakeholders’ perceptions of the market.

The GB market has gained significant attention as a sustainable investment instrument but faces challenges in scaling up to meet global sustainability goals [5]. Key barriers include a lack of harmonized standards, greenwashing risks, perceived higher costs for issuers, and limited supply [5]. Research has focused on market pricing, particularly the existence of a “greenium” or price premium for GBs [6]. Studies have also examined the economic and environmental effects of Green Bond financing, including stock market reactions and implications for stakeholders [6]. To address challenges, stakeholders must improve market efficiency, regulatory frameworks, and develop commonly acceptable evaluation metrics for ESG performance [7]

Recent systematic reviews and empirical studies have substantially advanced understanding of Green Bond markets, pricing dynamics, and investor behaviour. [8] synthesize over 70 empirical pricing studies, reporting an average negative *greenium* of about −12.44 basis points while highlighting significant regional and methodological differences in pricing results. Other reviews also document modest but context-dependent yield discounts for Green Bonds relative to conventional bonds, noting liquidity, certification, and reporting standards as important moderators [9]. Empirical research further shows how macroeconomic conditions and investor composition shape *greenium* dynamics, with retail investor demand and bond credibility playing measurable roles [10]. The literature also emphasizes persistent concerns about greenwashing and information asymmetry, with empirical evidence from China showing that selective environmental disclosure can elevate credit spreads and undermine pricing credibility [11]. Finally, work on regulatory frameworks and environmental commitments highlights that uneven disclosure regimes and market standards materially influence pricing outcomes and investor confidence [12].

Studies on the GB market have further highlighted the defining issue in the GB market [2,13–17]. Investment in GB carries the risk of greenwashing, as there is no proper mechanism for investors to ensure the appropriate use of the proceeds of their investments [17]. Moreover, the increasing number of green certificate providers in the GB market is giving rise to a so-called regulatory environment—one that is shaped by market practices rather than formal oversight—despite the absence of a proper legal framework, a governing body to monitor the market, or a commonly accepted definition of what constitutes a green certificate. According to [17], certificate providers aim to align the preferences of investors intending to participate in the Green Bond market with established green criteria, thereby enabling asset managers to implement those preferences efficiently. Local governments should focus on finding ways to give greater importance to environmental impacts in rating and certification systems by offering financial incentives and low-carbon subsidies [18].

Apart from different definitions, there are several instruments to measure the ‘greenness’ of projects, such as second opinions, green ratings, and GB indices, which are not consistent in terms of the degree of the scale, the availability of *ex-post* monitoring, and the availability of quantitative tools [19]. Hence, determining the true environmental impact—or “greenness”—of projects remains challenging, which in turn undermines credibility in the GB market and reduces investor confidence [20]. As argued in [21], many Green Bonds lack binding legal commitments to ensure that funds are directed toward environmental objectives. They highlight the disconnect between the marketed environmental promises and the actual legal structure of Green Bonds, which often mirrors that of conventional debt instruments. This accountability gap raises concerns about greenwashing, where issuers may overstate or misrepresent the environmental benefits of their bonds without significant legal or reputational consequences.

In addition to concerns about credibility, research has questioned whether Green Bonds offer compelling financial incentives to issuers. There is limited evidence of a consistent “greenium”—the premium that would manifest as lower yields for Green Bonds compared to similar Non-Green Bonds [22]. Their findings suggest that Green Bonds typically do not reduce the cost of capital for issuers, thus weakening a key rationale for adopting green financing. Without demonstrable financial advantages, firms may be less inclined to engage in Green Bond issuance, especially when faced with added reporting and verification costs.

Moreover, companies issuing Green Bonds tend to invest more in sustainable innovation and achieve stronger long-term returns. However, this effect appears concentrated among firms with established ESG credentials, indicating that Green Bonds may currently serve more as a signalling tool than as a mechanism for driving widespread corporate transformation [23].

The governance structure of the Green Bond market also presents obstacles. The absence of unified, mandatory regulations contributes to inconsistencies across jurisdictions, making it difficult for investors to compare bonds and trust the environmental claims attached to them. This regulatory fragmentation further complicates efforts to scale the market and build investor confidence [24].

As the Green Bond market continues to evolve, research should focus on its role in reshaping financial markets and fostering economic sustainability [25]. Thus, it is essential to identify how stakeholders perceive the market and what will provide assurance for investors and regulators regarding the green claims of corporate bond issuers [15]. Despite significant advances in understanding greenium dynamics, investor behavior, regulatory frameworks, and risks such as greenwashing, gaps remain in integrating these findings into cohesive conceptual models of market behaviour. Our study addresses these gaps by synthesising institutional barriers, stakeholder interpretations, and qualitative insights that complement existing quantitative research. The study’s main analysis is based on global publications, supplemented by interviews from the Australian and European markets. We suggest mechanisms to upscale the GB market to meet annual green investment needs worldwide, along with a theoretical framework that explains the relationship between stakeholder perception and scaling up the GB market.

This study contributes to the GB literature by integrating institutional theory and market failure theory into a unified analytical framework. Institutional theory illuminates how regulatory fragmentation, norm diffusion, and legitimacy pressures shape the evolving architecture of the GB ecosystem. Market failure theory, in contrast, explains how information asymmetries, externalities, and coordination failures distort pricing signals and investment incentives. By combining these perspectives, the study offers a distinctive interpretative lens that captures both structural-institutional constraints and market-level inefficiencies. This integrated framework enables a deeper understanding of why persistent barriers, such as definitional ambiguity, credibility concerns, and greenwashing risks, continue to impede the scaling of the GB market despite rapid global growth.

This paper is organized as follows. Section 2 discusses the theoretical overview, and Section 3 details the methodology in detail, alongside an explanation of the sample selection for data collection and the method of data analysis. Section 4 presents the study’s findings, and Section 5 concludes the paper.

## 2. Theoretical overview

This study explores how stakeholder theory can be integrated with green financing mechanisms, particularly in the context of the Green Bond (GB) market. The stakeholder theory of [26] defines stakeholders as any party who can affect or are affected by an organization’s outcomes. As a dominant framework in business ethics, stakeholder theory asserts that organizations should consider the interests of all stakeholders—not just shareholders—in their decision-making processes [27]. Rooted in the principles of corporate social responsibility, the theory emphasizes that long-term organizational success depends on delivering value to all primary stakeholders [28].

The theory further assumes that values are central to business and encourages managers to engage in shared value creation and cultivate meaningful stakeholder relationships [29]. Over time, stakeholder theory has evolved from its early conceptual foundations in the 1930s into a comprehensive ethical framework for analyzing corporate conduct [27,28]. Notably [30], advanced the theory in 1997 by introducing a typology for stakeholder identification and salience based on three key attributes: power, legitimacy, and urgency.

This theoretical lens offers a valuable tool for understanding and prioritizing diverse stakeholder interests, ultimately supporting sustainable practices and long-term value creation [28,30]. Our research adopts this core premise of stakeholder theory—that effective value creation involves acknowledging and responding to multiple stakeholder groups—to evaluate the success of Green Bond issuance through the lens of stakeholder perceptions.

Green financing, a central pillar of sustainability [31], is defined by [32] as “a credit intermediary of environmental protection’s capital” (pp 313). The integration of stakeholder theory and sustainability aims to safeguard the interests of stakeholders at both organizational and global levels. As such, examining how stakeholders perceive the Green Bond market is both timely and essential. Furthermore, the intersection of stakeholder theory and sustainability continues to offer fertile ground for theoretical development and practical application [33].

A vital instrument of the green finance market, GB, the primary concern of the current study, consists of three main stakeholders in addition to the government, namely: (1) investors, (2) issuers, and (3) policymakers (regulators). Investment in GB always carries the risk of greenwashing, as proper mechanisms do not exist for investors to ensure the appropriate use of their investment proceeds [17]

Therefore, by linking stakeholder perceptions with the green finance market, we suggest mechanisms to upscale the GB market to meet the annual green investment needs worldwide.

## 3. Materials and methods

### Data collection methods

We conducted a document analysis to gather data for this study. We further evaluated the information identified through the document analysis using interviews with investors, issuers and second-party opinion providers in the GB market to pursue convergence and justification and ensure internal validity. The analysis followed a structured qualitative coding process. First, all documents and interview transcripts were subjected to open coding to identify recurring concepts related to definitions of greenness, investor expectations, barriers, and policy recommendations. Second, axial coding was constructed to cluster related codes into higher-order thematic categories. Third, selective coding was employed to refine core themes and align them with stakeholder theory constructs (e.g., power, legitimacy, urgency).

Coding was constructed manually and iteratively, with themes continuously compared across document sources and interview transcripts. Discrepancies between documentary evidence and interview responses were examined to refine interpretations. Peer debriefing with two academic experts in sustainable finance and one industry practitioner further enhanced analytical reliability.

Rather than treating interviews and documents as equivalent datasets, the interviews functioned as a validation and contextual enrichment mechanism for themes identified through document analysis. Although interviews were conducted in 2018, the document review extended to 2021, allowing us to capture developments, structural and institutional features of the GB ecosystem, particularly definitional ambiguity, regulatory fragmentation, and credibility concerns, that continue to characterize market developments, thereby sustaining their contemporary relevance. Therefore, interview findings reflect stakeholder perceptions during and earlier phases of market development and may not fully represent post-2020 structural changes, such as EU Taxonomy or pandemic-related sustainable finance acceleration This dual approach enhances longitudinal validity, but we acknowledge the temporal difference as a limitation. Moreover, while the documentary analysis reflects global market developments, the interview sample is geographically concentrated in Australia and England. Therefore, the interview findings should be interpreted as exploratory insights rather than statistically representative global evidence. Their role is to validate and contextualize themes emerging from international documentary sources.

### Selection of the sample

#### Sample selection for document analysis.

Our study critically examined reports, periodicals and articles issued from 2007 to 2021 by different market participants and experts under four main themes identified through a critical examination of the literature. By doing so, we aimed to understand current GB market conditions, GB definitions, standards and criteria around the world, changes in the GB market, identified problems in the GB market, and recommendations to overcome these problems. Documents were selected based on their relevance to the Green Bond market, authoritative status, such as regulatory or institutional reports, and their influence on market practices or policies. This ensures that the analysis reflects both market-driven and policy-driven perspectives. The inclusion of indices such as the S&P Green Bond Index is particularly important as they capture market performance indicators that influence investor confidence. The reviewed documents under each theme are shown in Table 1.

**Table 1:** Themes and details of documents studied.

Theme/ Source	Primary purpose	Specific analytical contribution
**Theme #01 — Definition issue for ‘greenness’ in the GB market**	Clarifying standards of “greenness”	Highlighted how varying definitions create uncertainty, affecting issuer credibility and investor trust.
Green Bond Principles (ICMA, 2014–2021)	Voluntary global guidelines	Informed consistency challenges and the voluntary nature of disclosures
Climate Bonds Standards (CBI, Version 2.0)	Technical eligibility criteria	Provided evidence on lack of harmonization across standards
People’s Bank of China Announcement No. 39 (2015)	Regional regulatory framework	Illustrated differences between Western and Asian interpretations of “green”.
IISD Report by Kidney & Oliver (2014)	Early framing of green finance	Identified definitional disputes that persist across markets
**Theme #02 — Investors’ expectations from the GB market**	Investor motivations and trust	Showed how expectations shape demand and pricing
World Bank Reports on GB issues (2013–2021)	Sovereign and MDB issuances	Reflected investor appetite for credible, large-scale issuances
Second Opinion on the World Bank’s GB Framework (2015)	External review mechanism	Highlighted importance of credibility tools (second opinions)
Investor Statement on GBs & Climate Bonds (CBI, 2014)	Collective investor voice	Stressed demand for transparency and accountability
Ceres Investor Network on Climate Risk (nd)	Investor advocacy	Showed long-term expectations for impact reporting
Green Bond Symposium Summary (World Bank, 2013)	Market-building dialogue	Provided insights into early investor concerns
**Theme #03 — Other obstacles to scaling up the GB market**	Market barriers	Identified gaps in policy, liquidity, and transaction costs
OECD & Bloomberg Report (2016)	Policy–finance linkage	Highlighted structural barriers needing public–private coordination
Jun, Kidney & Pfaff (2016)	Country experiences	Identified local institutional constraints in emerging markets
EU Study on Resource-Efficient Investments (2016)	Sectoral financing	Showed challenges in linking GBs to environmental impact
CBI Consultation Paper (2015)	Role of government	Outlined need for policy support to scale GB markets
CBI State of Market Report — China GB Market (2015)	Country case study	Demonstrated rapid but inconsistent market development
**Theme #04 — Recommendations to overcome challenges in the GB market**	Proposed solutions	Helped refine study’s policy recommendations
All documents reviewed	Market benchmarking	Used to examine credibility, liquidity, and pricing issues
S&P Green Bond Index (2014)	Market performance	Reflected issuance volumes and investor confidence
Bloomberg Barclays MSCI Global GB Index (2014)	Global tracking	Provided comparative insights across geographies
Moody’s GB Assessment Methodology (2016)	Credit risk evaluation	Highlighted rating methodologies as barriers/opportunities
CICERO Framework for Second Opinions (2016)	External validation	Demonstrated importance of credibility tools
HR’s Green Bond Rating Methodology (2017)	Alternative rating	Showed diversity of assessment approaches creating fragmentation

#### Sample selection for interviews.

The interviews were designed to complement the document analysis by validating emergent themes rather than to achieve statistical generalizability. While the sample is geographically limited, the participants were selected for their expertise in the Green Bond market. A small sample was employed accordingly to provide suggestive and supportive evidence to the study’s main findings using purposive sampling, based on existing social groups of people who knew each other through working or socialising together and knew the GB market. A purposive expert sampling strategy was adopted to ensure that participants possessed direct and substantial involvement in the GB market. Rather than aiming for statistical generalizability, the objective was to obtain information-rich cases capable of providing deep insights into institutional practices, regulatory interpretations, and investor expectations. Participants were selected based on three criteria: (1) executive-level decision-making involvement in GB issuance, investment, or certification; (2) direct engagement with GB frameworks (e.g. GBPs or CBS); and (3) representation of key stakeholder categories identified in stakeholder theory; issuers, investors, and external reviewers. The final sample of five experts aligns with qualitative research norms for elite interviews, where depth of expertise compensates for smaller sample sizes. The interviews were designed to validate and critically examine themes emerging from the document analysis rather than to generate statistically representative findings. The most appropriate person to be interviewed from each organisation was first identified through telephone calls. The consent to participate has been obtained by emailing the Plain Language Information Sheet (PLIS) to the interviewees. PLIS contains the researchers’ profiles, research objectives, support service lifeline contacts, data security, and the choice to accept or reject participation with the consent form. Upon receiving the email confirmation to participate, the interviews were conducted from 1^st^ May 2018–31^st^ May 2018. This study received ethical approval from Federation University Australia (Approval No. B17-079). All participants were aged above 30 and executive-level employees who represented the following sectors:

- GB issuers (Australia)—executive-level employees of issuing organisations
- GB investors (Australia)—individual investors and institutional investors (executive level)
- Second-party opinion providers (Australia and England – CICERO/ ICMA/ CBI).

We contacted five people who represented investor, issuer and second-party opinion provider institutions for the interviews. Two people were interviewed through a group discussion, while the others were contacted through online interviews. This allowed us sufficient opportunity for numerous discussions, negotiations and comments among the participants within the limited time of the interviews. The interviews were approximately 30 minutes in duration. A venue for the group discussion was selected based on convenience to the participants. In addition to audio recording, note-taking was also conducted. Audio recordings were transcribed verbatim with the consent of participants for data analysis purposes.

At the end of the discussion, participants were given permission to re-contact them in the future for further information and clarification, if required. Accordingly, the interview transcript was sent to all interviewees for their approval. Participants were asked to review the transcript and make any changes if desired. Approvals for transcripts were received with minimal modifications to the original transcripts.

Thematic saturation was assessed iteratively during data collection and analysis. After the fourth interview, no substantively new themes emerged beyond those identified through document analysis. The fifth interview was conducted to confirm consistency and ensure adequate representation across stakeholder categories. Given the focused scope of inquiry and the homogeneity of expertise among participants, thematic redundancy was observed, suggesting that saturation at the level of key analytical categories had been reached.

### Inclusivity in global research

Additional information regarding the ethical, cultural, and scientific considerations specific to inclusivity in global research is included in the Supporting Information.

## 4. Data analysis and findings

The document review is instrumental in purifying concepts, identifying theoretical boundaries, and locating the fit and relevance of categories [34]. Therefore, when interviewees presented new views, the documents were re-analysed to determine the presence of those opinions as new insights into the GB market. Moreover, we were satisfied with the completion of the data collection and analysis process only once all necessary proof from the documents and interviews reflected a consistent picture of the stakeholder perceptions of the GB market.

Our findings are not only consistent with prior literature but also add novelty by synthesizing evidence from both stakeholder interviews and authoritative documents, thereby revealing the persistence of barriers despite extensive policy discourse.

The analysis is grounded in Institutional Theory, which highlights the role of norms and regulatory pressures, and Market Failure Theory, which explains the persistence of inefficiencies requiring government intervention. Together, these frameworks provide a robust lens to interpret barriers in the Green Bond market. Rather than merely summarizing themes, we critically evaluated them through the lens of institutional pressures and market inefficiencies, showing how recurring structural issues continue to hinder scalability.

### Triangulation strategy

Triangulation was implemented at three levels:

(1) Data-source triangulation – comparing institutional reports, regulatory publications, and interview evidence;
(2) Stakeholder triangulation – examining perspectives from issuers, investors, and second-party opinion providers;
(3) Theory triangulation – interpreting findings through stakeholder theory, institutional theory and market failure theory

Convergence across these sources enhanced internal validity, while areas of divergence were explicitly acknowledged and analytically discussed.

### Data transparency and audit trail

Due to ethical approval conditions (Federation University Australia, Approval no. B17-079), full interview transcripts cannot be publicly disclosed. However, anonymised excerpts are included in the manuscript (next section) to illustrate interpretive grounding.

## Findings

To enhance transparency and demonstrate how interpretations are grounded in empirical material, representative anonymised excerpts from interviews are presented alongside document evidence. These excerpts illustrate how stakeholder perspectives informed theme development. Moreover, while the primary analytical foundation of this study is institutional document analysis, interviews functioned as a validation mechanism and contextual enrichment tool. Therefore, conclusions reflect convergence between documentary patterns and stakeholder perspectives rather than relying solely on interview testimony. The themes identified in the 2018 interviews remain observable in subsequent institutional reports up to 2021, reinforcing the persistence of these structural barriers.

Table 2 illustrates the coding progression from first-order empirical statements (derived directly from interview transcripts and documents) to second-order analytical categories and aggregative themes. This structured approach enhances transparency in how interpretations were derived.

**Table 2:** Coding structure and theme development.

First-order codes	Second-order codes	Aggregate themes
“lack of common definition”	Definitional inconsistency	Definition problem
“too many country specific”	Regulatory fragmentation	
“high reporting burden:	Administrative cost	Market barriers
“overpricing issue”	Pricing mismatch	
“need government backing”	Policy intervention demand	Policy solutions
“we do our own due diligence”	Investor verification requirement	Investor expectations

Interview excerpts included in the findings were selected based on their representativeness of recurring themes rather than rhetorical strength. Quotes were anonymised and labelled as Respondents 1–5 in accordance with ethical approval requirements.

### Theme # 01: Definition issue for greenness in the GB market

The findings suggest that perceived credibility gaps persist in certain segments of the global GB market, particularly in relation to definitional harmonization and reporting transparency As there are several definitions of GBs in circulation, it would be ineffective to list all the available definitions. However, we explored the features commonly used to identify a GB in the capital market and the areas that remain unclear and require clarification.

Generally, GBs are defined as fixed-income securities that raise capital for a project with specific environmental benefits [35]. A GB only differs from a normal bond regarding its proceeds allocation. All available definitions for GBs highlight the fact that proceeds from issuing a GB should be invested in ‘green’ projects. Therefore, the major problem in GB definitions is the evaluation of ‘greenness’. As identified in Inderst, Kaminker and Steward in [14], measuring greenness as a common problem in any type of green investment and a problem that is not limited to institutional investments. In explaining the main problem of defining ‘green’, they emphasised that:

*Definitions of ‘green’ can be explicit or implicit. Some are very broad and generic; others are more technical and specific. Some are investment-driven; others come out of ecological or ethical discussions. The greenness of assets can be postulated in absolute terms (a good or technology is green or not green) or in relative terms (e.g., one company has lower greenhouse gas emissions than another or is more energy-efficient).* (P.10)

In reference to GBs, the same problem prevails, as different economic and policy areas identify ‘greenness’ differently. Experts in the GB market stress that GB market growth is ‘strangled’ because of the presence of less-harmonised bond frameworks [3]. Climate Bonds Initiative and the International Capital Markets Association act as the central regulatory bodies for GB issues. While the ICMA defines GBs in line with the GBPs, the CBI issues a certificate for greenness to label a bond as a GB. The ICMA provided the same definition in the GBPs issued from 2014 to 2021, yet changes were made to the list of eligible green projects. The GBPs of the ICMA do not broadly discuss ‘greenness’ but provide broader categories of eligible green projects. The GBPs illustrate that new areas are introduced as ‘green’ each year. As the secretary to the GBPs, the [36] defines a GB as:

*Any type of bond instrument where the proceeds will be exclusively applied to finance or re-finance in part or in complete new and/or existing eligible green projects and which follows the four Green Bond principles*. (p.2)

In other words, a bond is a GB if the issuer uses the proceeds of the issue totally for capital expenditures for green or climate-related environmental benefits [37] and follows all four GBPs. The two critical aspects emphasised by this definition are financing and traceability, which are vital to the development of the GB market. GBs that provide investors with access to the market for information on ongoing projects that meet the green standards grant some assurance to investors through traceability and reporting requirements [38]. However, the [39] criticised the space provided to re-label a Conventional Bond (CB) as a GB through this definition. It argued that this might provide an incentive to ‘greenwash’. Greenwashing is identified as giving the impression of changing a project to have environmental benefits, while not actually making this change [19]. However, Urik Ross, the global head of public sector and sustainable finance at HSBC, stated that:

*I don’t think an isolated greenwashing incident would be enough to derail the growth of the market. If this happened, it would be very damaging for the reputation of the issuer. But the market is now sufficiently mature to withstand a setback of this kind.* [40] (p.32)

However, there are no sector-specific or common criteria to assess the compliance of projects with GBPs, and the issuer is entitled to label their projects as green upon the issuance of GBs. Nevertheless, to enhance the credibility of the issue, issuers can obtain a green label through the CBI. To assess the ‘greenness’ of projects, the CBI can assess its compliance with the GBPs using the CBS. However, the criteria to assess greenness under the CBS are currently only available on projects categorised as solar energy, wind energy, geothermal energy, low-carbon transport or low-carbon building [41]. The CBS does not define GBs; however, if a GB meets the requirements of the CBS, it can be identified as a certified climate bond.

According to [15], GBs are:

*Bonds or debt securities specifically issued to finance environmental protection, sustainability or specific climate mitigation and adaptation measures.* (p.8)

This characterisation is only a general identification of what is meant by GB, based on the ICMA definition. While most issuer organisations use the definition and procedure provided by the ICMA through the GBP, the World Bank Group (the bank and IFC) uses a different characterisation for GBs. A general issuer in the GB market issues bonds to raise funds for its green projects. However, the World Bank Group uses the GB proceeds for lending to climate-friendly institutions or governments in different countries that require funds for projects that are identified as either mitigating or adapting to climate change [42]. According to *The World Bank* [43], a Green Bond is defined as:

*A debt security is issued to raise capital especially to support climate-related or environmental projects.* (p.8)

The World Bank encourages issuers to seek third-party review of their GBs from the CICERO at the University of Oslo, which is considered a notable development in the GB market [15]. When evaluating the World Bank framework, the CICERO stated that:

*We find no obvious weaknesses in the World Bank framework as it now stands* [44] (p.6)

The [42] claims that the rigorous process it follows for GB issues involves rating its GBs as Aaa/AAA (Moody’s/S&P). This reflects the financial reliability associated with the World Bank GBs and can be identified as a good sign for investors to satisfy financial credibility. Though it does not validate the bond’s environmental performance, the World Bank group has collected more than US$16 billion in over 200 GB issues since 2008 to assist with climate- and environment-related investments. This includes more than 185 GBs in 23 currencies, worth approximately US$16 billion equivalent by the World Bank [45], and 90 bonds worth US$7.25 billion across 12 currencies by the IFC [46].

The two main definitions discussed above—the ICMA definition and the World Bank definition—are internationally accepted and used by different issuer institutions worldwide to recognise their projects as green when issuing GBs for project financing. For the first time in the world capital market, China has characterised a GB in a manner suited to its local market. The PBoC defined GBs through its *Announcement No. 39 (2015)* as:

*The securities issued by financial institutions in accordance with law, with a purpose to raise funds to support the green industry and to repay capital with interests by contracts.* [47] (p.1)

However, this definition is only used in the interbank market, and has subsequently been criticised as a local definition that cannot be applied to international bond issues or to local issues by any other sector [48]. The 2016 China GB Market report issued by the CBI emphasised that this definition and the projects it identifies as green are not consistent with international standards, and are not considered by international investors. Moreover, the project categories and process for issuing a GB according to the PBoC guidelines and NDRC regulations are also not synchronised. However, these guidelines have attracted a significant investor base as mandatory requirements. As a result, China became the largest GB market in the world in 2017 [49] and was second in 2018 [50].

Although the SEBI of India has issued country-specific guidelines for GB issues, they consider the harmonisation of the ‘green’ definition with international standards an essential factor to ensure international investors are comfortable with their GB issues. Accordingly, the SEBI recommended that their issuers use the CBS to identify the ‘greenness’ of their projects [51].

Moreover, by 2021, many jurisdictions, including the European Union, China, Mongolia, the Russian Federation, Mexico and South Africa, have developed Green Taxonomies in order to make participants aware of what really could be counted as green [52].

While international and local authorities define GBs in terms of eligible project categories, international green indices and second-party opinion providers use their own methodologies to identify GBs. The S&P Dow Jones Indices introduced the S&P Green Bond Index in July 2014, to increase transparency in relation to the features of the GB market and enhance the quality of reporting and tracking the use of proceeds. The index only considers GBs labelled as ‘green’ by the issuer and certified by the CBI [53]. identifies a GB as:

*A bond whose proceeds are used to finance environmentally friendly projects.*

The Barclays/MSCI Global Green Bond Index (GGBI), which was created in November 2014, defines GBs as:

*Fixed-income securities in which the proceeds will be exclusively and formally applied to projects or activities that promote climate or other environmental sustainability purposes through their use of proceeds* [54] (p.3).

For a bond to qualify as a GB in the GGBI, at least 90% of proceeds must be used for either new or existing environmental projects in six broad categories. Both self-labelled and unlabelled bonds are independently reviewed by MSCI’s ESG research in line with the four GBPs of the ICMA to determine whether they can be recognised as a GB. Even if the bond under consideration is not explicitly sold as green, it can be considered for the index.

Moody’s Investor Service has published a more systematic methodology to assess GBs, called the ‘Green Bond Assessment’ (GBA) methodology in 2016. Rather than acting as a credit rating, GBA provides an opinion on the effectiveness of the issuer’s process for managing, administering and allocating proceeds and reporting on environmental projects. The GBA [55] defines GBs as:

*Fixed-income securities, both taxable and tax-exempt, that raise capital for use in projects or activities with specific climate or environmental sustainability purposes. These include debt obligations with direct resource to issuers, project finance or revenue bonds, with and without recourse to issuers, and securitizations that collateralize projects or assets whose cash flows provide the first source of repayment.* (p.02)

The Green Bond Rating Methodology (GBRM) was published by HR Ratings in Mexico, and has been effective since 31 January 2017. The rating provides a scale similar to Moody’s GBA methodology to categorise projects financed through GBs. In developing the evaluation criteria, the GBRM considered both GBPs and CBS, as they provide internationally accepted guidelines. The GBRM defines a GB as:

*any type of fixed income asset whose proceeds are used to finance or refinance, in part or in full, new and/or existing environmentally sustainable projects.* [56] (2017, p.2)

As a second-opinion provider in the GB market, the [44] defines a GB as:

*A fixed-income financial instrument issued with the aim of financing climate or environmentally friendly projects. (p.02)*

Further explaining the role of GBs, the CICERO emphasises that a GB should be able to lower adverse environmental consequences and reduce carbon emissions by funding mitigation and adaptation projects and technologies. Thus, the ability to contribute to the mitigation of carbon emissions and adaptation of eco-friendly actions is recognised as a prerequisite of a GB.

All definitions discussed above emphasise that the type of investment or project should be climate-related, environmental or ‘green’. The issuer of the bond uses different definitions of GB to suit their requirements and business profile [15,43]. However, to enable high reliance in the market, it is essential to avoid different interpretations of what is meant by ‘green’ [15]. Furthermore, it is evident that great efforts have already been invested by different institutions to define ‘green’ in relation to GBs. However, none of these definitions are able to precisely quantify the green measurement criteria in their definitions, except for Moody’s GBA methodology. When explaining the need to ensure straightforward and clearer guidelines in the GB market, Sean Kidney stressed that regulators should clarify the projects eligible to be labelled as green, thereby enabling investors to assess the positive effects of their investments:

*A vast gas plant in Nigeria would not qualify as a green project, whereas clean energy projects around the world do; manufacturing a hybrid hummer should not qualify, whereas electric cars should, where you put them* [3].

As a method of validating the results, we conducted interviews with two institutional investors, one institutional issuer and two institutional external reviewers (second-party opinion providers). The nature and principles of GBs that underpin the major findings obtained from the review of the documents were discussed. The two investors, one issuer and two external reviewers were identified as Respondents 1, 2, 3, 4 and 5, respectively. These five respondents were not provided with the findings of the document analysis in our study. Instead, questions were asked to obtain their independent opinions of the nature and operation of the GB market.

All respondents used the GBPs and criteria issued by the CBI through the CBS as the basis for defining GBs and identifying a project as eco-friendly. When answering the question concerning how they defined a GB, they all highlighted the GBPs as their base guideline:

*I have never looked at our own definition of GBs. We typically look at what is brought to us by our issuer. We have dealt with only a limited number of issuers, like Australian banks, supplies by our treasury, Queensland Treasury and the World Bank, and we look at what they are proposing and then confirm that it does appear to be consistent with what we would expect the GB to make. So we would expect it to be delivering some form of environmental benefits, so that’s our simple definition, and it just so happens that all of the GBs we’ve invested in comply with the Green Bond Principles.*

(Respondent 1)

*We have selected the CBI as the independent non-governmental organisation to rate our GB issuances. We define GBs as having independent certification as meeting the appropriate criteria, and all GB issuances by [Name of the Institute] will be assessed using the CBI framework.*

(Respondent 3)

However, Respondent 3 expressed the view that while GBPs are essential for up scaling the market, ensuring market credibility and an independent certification are not guaranteed. Furthermore, it is difficult to match certain environmental resilience projects with the CBI framework at times because of the absence of criteria in the CBS and GBPs for some industries:

*We follow the Green Bond Principles, but take a deeper dive into the definition of green based on international climate research.*

(Respondent 4)

*We use the International Capital Market Association’s definition.*

(Respondent 5)

However, the investors emphasised that market participants must conduct their own due diligence. This is most important because of low standardisation and the possibility for greenwashing in particular GBs. They highlighted that most bond issues seem to comply with the GBPs at present. However, they were still required to assess them case by case, until they were comfortable that the underlying projects were truly green.

In line with the issues identified through the document review, the introduction of country-specific regulations was recognised as problematic because of the discrepancy with international standards. Elaborating on this concern, the external reviewers made the following observations:

*too many country-specifics will be complicated—but there are lots of actors to consider, e.g., stock exchanges are inputting their own requirements now, also China and India’s governments. ICMA could help with a mapping of international requirements to guide issuers.*

(Respondent 4)

*Harmonisation should happen over time.*

(Respondent 5)

Such opinions of stakeholders provide additional support for the themes emerging from the document analysis. These views demonstrate that, despite the GBPs and CBS providing guidelines to the GB market, low standardisation still reduces market credibility. Moreover, harmonisation with international regulations is essential to bring local markets to the required scale.

### Theme # 02: Investors’ expectations from the GB market

Sean Kidney, the CEO of the CBI, emphasised that the feasibility of green financing can be confirmed to investors through appropriately structured GBs [3]. However, it is essential to consider which outcomes investors expect from GB investing. Therefore, aside from addressing the issue of defining ‘greenness’, this study also analysed documents, as mentioned in Table 1, to identify investors’ expectations of the GB market.

The World Bank summarised the key concerns of investors in the GB market in 2013. A symposium was conducted to share the different perspectives of GB issuers, investors and intermediaries for the benefit of the GB market. Investors emphasised four main points as the key considerations when making GB investment decisions [57] (p.02).

*1. Transparent disclosure of definitions and processes by issuers, combined with strong governance and impact reporting, is paramount*

Although all institutional reports that discuss barriers in the GB market emphasise the definition issue, investors who participated in the Green Bond Symposium hosted by the World Bank Treasury did not require a rigid definition to assess ‘greenness’. Instead, they required high transparency by issuers during the GB issuing process and reporting. Irrespective of the definition given by issuers, investors undertook an assessment to determine whether the proposed project suited their green portfolio using investor-specific criteria. Therefore, a strong governance procedure or third-party verification was required for investors to be satisfied with the transparency of the disclosure of definitions provided by the issuer.

Validating the above facts, the interviewees of our study, who represented investor institutions, also expressed the same concern as an investor expectation:

*I think in terms of the fact that these differences in definition isn’t really an issue, we can say for us because we are not investing because of the technicality.*

(Respondent 2)

*We do our own analysis and also, speaking of we are, we also ideally watch the issuer to be reporting on the benefits of that project and time, so we want that transparency, like a positive impact in the future, so that’s really important to us. So that’s something we look at before investing as well, so the prior examples of how they reported or how they propose to report, so the way they have confidence that the green project is actually delivering a benefit.*

(Respondent 1)

*2. Financial characteristics of GB products are the determining factor for an investor to participate in a transaction*

The determinants of a decision in relation to CBs—such as the risk–return profile, size, currency, maturity, expected liquidity and structure—were also treated by symposium participants as the prerequisites for GB investment decisions. While some investors expected the same level of financial return from a GB as from a CB from the same issuer, some investors focused more on GB pricing. They expected different pricing from the same issuer for GBs and CBs of similar size and maturity. From a Market Failure perspective, this absence of a consistent “greenium” weakens financial incentives, limiting voluntary market expansion without policy intervention.

*3. The expected level of external ‘comfort’ required depends on the type of issuer and product*

The investors emphasised that they required transparent disclosures of the earmarking process, compliance procedure, investor engagement and third-party certification to minimise the risk of investments in green assets through GBs. They expected high confirmations from corporate issuers and on new green products to ensure credibility. Additional confirmation for credibility was not required from supranational issuers and other issuers who were reputed for eco-friendly projects. In contrast, the symposium participants who were not investors highlighted that requiring mandatory third-party verification would increase the issuance cost of GBs.

*4. Recognition that this as an ‘evolution not a revolution’*

The main objective of the GB market is accepted to be to ‘*mobilize finance for environmental challenges at scale*’ [57] (p.02). Investors accepted that market standards should be gradually developed, with standardised formats for reporting. Therefore, investors did not expect immediate action. The investors, issuers and intermediaries all agreed that the solutions should be implemented step by step, with simple and clear standards and definitions. Moreover, they did not expect one common definition to cover all green areas, but required strong environmental and social safeguard policies with criteria to assess green impacts. The symposium concluded with the agreement that the GB market should ‘*not allow perfect to be the enemy of good*’.

The CBI issued investor statements in 2014 in relation to GBs and climate bonds, with 19 signatories who represented asset owners, investment managers and individual funds that managed a combined asset worth US$2.62 trillion around the world. They expressed interest in contributing to the GB market as responsible parties to overcome environmental challenges. In the statement, they emphasised that:

*We, as investors and fiduciaries, understand that we have a responsibility to address threats to the future performance of our investments from climate change as well as a responsibility to secure our clients’ savings through sustainable and responsible investments. We believe Green Bonds can be part of our strategy to accomplish both these aims* [58] (2014, p.1)

The signatories explained their common expectations from the GB market to reach the required market scale by meeting the risk–return trade-off of investors. They emphasized the importance of government intervention, developing clear and industry-specific standards, and ensuring the transparency of the use of proceeds and their effects, in order to scale up the GB market.

The INCR offered directions to issuers in the GB market regarding investor expectations of GB-supported projects. Twenty-seven investors worldwide signed the statement, expecting a common framework for investors, issuers and underwriters of GBs. They accepted the GBPs of the ICMA as essential guidelines to scale up the GB market, along with a few further developments. They expected high consistency in standards and guidelines to enable better comparison and high transparency. Accordingly, the [59] addressed four key issues in the market from the investor’s perspective, namely: (1) Eligibility: general criteria for green projects, (2) Initial disclosures and intended use of proceeds, (3) Reporting on use of proceeds and project effects/benefits, and (4) Independent assurance.

Accordingly, investors expected further criteria to minimise greenwashing concerns and increase the credibility of a GB issue. They believed that the project categories identified by the GBPs to be climate and environmentally friendly. However, issuers were expected to compare their targeted environmental impacts with publicly available criteria to improve the credibility of a particular issue. Moreover, the investors required additional due diligence reports from projects that offer only marginal benefits to the environment, despite being originally categorised as ‘green’, such as large-scale hydro and nuclear power generation, and greenhouse gas reductions from coal-fired power plants. Further, for projects that are not originally environment and climate focused, investors require a substantial contribution to the sustainable management of eco-resources, plans to reduce waste or pollution, and enhancements to environmental quality.

Prior to issuance of a GB, issuers were required to disclose the proposed project categories, framework used to select the projects to finance through GBs, criteria for environmental impact assessments and expected environmental impacts of projects. Investors expected issuers to provide this information in a separate document, enabling them to compare the information among issuers. In addition, investors expected certain prerequisites from proposed projects, as follows:

- Using environmental studies, internal research and third-party assessments, projects should exhibit clear environmental benefits that can be evaluated, defined and (when possible) quantified
- Investors should receive information about the percentage of bond proceeds in between project financing and refinancing
- Investors should receive an explanation about the management process to track the use of proceeds
- The proceeds from GBs should be fully allocated to projects within a reasonable timeframe, probably within the term of bond maturity
- Issuers should explain the reporting procedure to investors, with timeframes and the mode of communication

The investors are also required to report the impact annually from GB issuers, along with information about the use of proceeds. The [59] requested early issuers in the GB market to initiate the development of standards for impact reporting.

Moreover, investors expected second-party opinions on the use of proceeds from climate and ESG experts and financial auditors. The investors believed these opinions would help them feel more confident about project selection, environmental impacts, issuers’ governance and monitoring guidelines, and issuers’ capacity to meet reporting requirements. While the investors strongly emphasised impact reporting, the issuers of GBs stressed the negative consequences of complicated reporting requirements.

Opinions of GB issuers and regulators in the market at the Asia Securities Industry and Financial Markets Association (ASIFMA) Green Finance Conference, organised by the United Nations Climate Change Secretariat and United Nations Economic and Social Commission for Asia and the Pacific in July 2018, are quoted by [3]. Sean Kidney expressed his view on the unnecessary requirements in some reporting frameworks, which complicate the issuing procedure. He stated that it is not necessary to implement compulsory reporting on greenhouse gas emissions for solar energy projects, while showing the necessity of reporting this measure by geothermal projects. He explained that it is necessary to minimise the work of the issuance, while maximising the work of the wider framework in which GBs are issued.

The following quotations from GB issuers indicate the ways in which different regulations and standards by different institutions increase the complications involved in the GB issuing procedure:

*We are strangled by all the standards around Green Bonds, and when an opportunity comes, we cannot move forward.* (Caroline Waqabaca, Chief Manager of Financial Markets—Reserve Bank of Fiji)

*Corporations have a lot on their plates. Spending five weeks with an auditor is not something any corporation would like to do. Green Bond standards and requirements can be simplified a bit in order to fast-track the development and the adoption here.* (Esther An, Chief Sustainability Officer—City Development Limited)

These two statements highlight the burden on issuers to prove the trustworthiness of their GB issues to investors and analysts. This observation reflects institutional pressures described by Institutional Theory, where compliance with evolving norms and standards increases organizational complexity and resource burdens.

Supporting the findings of the document analysis, the interviewees of our study stressed that they would like to invest in Australian dollar-denominated bonds to avoid currency risk. When discussing other factors considered by investors, they emphasised the nature of the issuer’s general business, reputation, liquidity and risk–return profile:

*I guess an additional set of criteria into these other effects that we don’t just look at the specific project, but we also look at the organisation that is developing that project and make sure that it doesn’t have any fossil fuels operations, because, as it was said, those projects go to the Green Bonds account of our sustainable options, which have fossil fuel screens [so] we need to be really certain that there is no adverse consequences or as some adverse associations.*

(Respondent 1)

*because there is a Green Bond, say, as a sustainable option, we met, for example, we don’t want to invest in a wind farm where it’s potentially run, say, by a company which could be considered inconsistent with what we have in our sustainable options need to be.*

(Respondent 2)

*Because they exploit fossil fuels.*

(Respondent 1, explaining the view of Respondent 2)

The document analysis revealed that the market participants expected price differentiation between GBs and CBs. Confirming this finding, two representatives of investor institutions explained that they would not invest in GBs if they were not priced appropriately. Particularly with GBs, they valued both ESG and price considerations. Even if they were comfortable with the ESG side of the project, if the price did not match what they were willing to pay, they would not invest. The same concern was further confirmed by Respondent 3, who explained that despite the expectation to have a price difference, there was no such pricing benefit from the issue of GBs:

*the pricing of the GB was also the same as traditional bonds. There was no pricing benefit as was expected.*

(Respondent 3)

Additionally, the profitability of GBs and the ability to repay their investment (that is, the default risk) were also considered by investors:

*Absolutely, the project’s profitability is the first criterion that the fixed-income team look at before they do due diligence. They’ve become comfortable with the investment characteristics of the bond issue, the way we’ve been looking at and the way we’ve been comfortable with. Then they come into the due diligence, and we would never invest in a Green Bond project if the investment did not stack up.*

(Respondent 1)

*Also, in the debt market, profitability is not a focus—it’s repaying your debt … They don’t have to make money. They just have to repay their debt.*

(Respondent 2)

In addition, the two respondents explained that government intervention in the market is of high importance:

*Potentially, they might not be hugely profitable, but if it is being backed by a government, in the case of the World Bank ones, it’s been backed by the local council or whatever it is, and then they can make repayments on those loans, then that sort of thing is important to us—it’s not that they make money.*

(Respondent 2)

They emphasised that they would prefer to invest in GB issues by the treasury, financial institutions or the World Bank. These explanations from investors confirmed the findings of the document analysis regarding investors’ expectations of the GB market.

### Theme # 03: Other obstacles for scaling up the GB market

In addition to the absence of a commonly accepted green definition in the GB market, other problems have been identified by different institutional and national reports.

In the IISD report [15], suggested three major barriers in the GB market: (1) lower market credibility because of the absence of a clear definition, (2) lower project transparency and (3) liquidity and risk/return constraints. According to the IISD report, the GB market has a significant potential institutional investor base who are seeking clear green features for investments towards sustainability goals. Two hundred and fifty-nine institutional investors who possess a combined US$29 trillion in assets contributed to the global investor coalition on climate change. Although they have the intention to contribute to a low-carbon economy, the absence of transparent green claims prevents their investment. Furthermore, lower market liquidity due to the lack of large-sized GB issues and low credit ratings also poses a challenge in the market. In relation to zero-emission renewable energy projects, less than 50% from the total outstanding GBs achieved investment grade.

The consultation report issued by the [60] in 2015 identified the prevailing problems under five themes: (1) challenges to market development, (2) challenges to increasing GB issuance, (3) challenges to enabling a wider range of issuers and projects, (4) challenges to increasing GB investments and (5) challenges to increasing cooperation in scaling the GB market. First, the absence of clear standards and guidance was identified as the main problem preventing GB market growth. The report emphasised the need to implement well-established green credentials in the GB market to achieve climate goals worldwide. It argued that the problem of using GB proceeds to finance projects with little environmental benefits—and claiming these projects to be green (greenwashing)—can only be mitigated through sound standards and policies. Second, limited bankable green projects and reduced awareness among potential green project owners about the benefits of financing these projects through GBs are highlighted as the main challenges to increasing the supply side of the market. Lack of awareness is a problem mainly in emerging economies, as evidenced by the current list of GB issuers, who largely represent developed economies.

Third, bond diversity and scale were identified as major factors to be considered in GB market development, as greater scale and diversity will help attract more issuers and investors to the market. Therefore, small-scale projects, lack of aggregation mechanisms and low credit ratings were introduced as the major challenges to enabling a wider range of issuers and projects. Fourth, the report identified capacity constraints because of a lack of publicly available historical data, lower availability of credit ratings and reduced awareness of investors as challenges to increasing GB investments. Finally, lower harmonisation and cooperation between key market participants were identified as the main challenges to overcome. Overall, government intervention was highly recommended by the CBI [60] as a remedy to the identified key barriers. The report suggested some recommendations/policy actions to be taken by the public sector, which are discussed under Theme 04 of this study.

The CBI [61] identified a number of common barriers to GB market growth. It argued that strong intervention and push by policymakers and regulators will help overcome these issues. The OECD’s report also identified the definition issue as a major obstacle. The identified barriers were as follows (p.20):

- Lack of infrastructure projects corresponding to a long-term governmental commitment to low-carbon development
- Lack of commonly accepted green definitions
- Investors with limited capacity to analyse green investments
- Scale and mismatch among projects, bonds and institutional investors
- A lack of suitable aggregation mechanisms—non-standardised projects and cash flow instability
- Low credit ratings for potential GB issuers and green projects, especially in emerging economies.

These barriers in support of the G20 Green Finance Study Group (GFSG) by [16]. The GFSG conducted a survey to identify the barriers hindering GB market development, using 24 investors, issuers and intermediaries in the GB market. Although the results were based on local markets, these barriers could be identified as general challenges to the GB market worldwide, though the importance of each factor will vary in different markets. The survey results are reported in terms of importance in Table 3.

**Table 3:** GFSG survey results.

Identified problem	% of responses
Lack of awareness of the benefits of GBs and existing international guidelines and standards	74
Difficulties for international investors to access local markets	67
Lack of domestic green investors	59
Lack of GB ratings, indices and listings	56
Lack of targeted incentives for GB issuers	55
Lack of local GB definition and guidelines	43
High cost of meeting GB requirements	41

Additionally, the survey results identified the lack of supply of labelled GBs in some markets as a problem to overcome. Further, the unavailability of a reliable tool to differentiate green assets and non-green assets was identified as another main reason for low GB investments.

In addition to the common factors identified by the reports discussed above, survey results indicated a major barrier from the issuer’s perspective: the high cost of meeting GB requirements. The findings of the document analysis strongly suggested the low market attraction by institutional investors due to low credibility as the main problem in the GB market. However, the reports emphasised that there is a considerable potential investor base for the market if the market ensures there is no greenwashing. Therefore, it is important to examine the reason for the low supply of labelled GBs in the market. Hence, identifying the high cost of meeting green credentials is worth further discussion.

To improve trustworthiness, it is essential to verify the greenness of projects and to continuously monitor and report the use of proceeds. However, at this stage, there is no commonly accepted definition, standard or methodology for this assessment and reporting; thus, potential issuers must seek professional services from external reviewers for pre- and post-audits. According to [16], the professional service fee can range from US$10,000 to US$100,000 per annum. Additionally, issuers must spend significant amounts on periodic reporting requirements. This high cost discourages potential issuers from issuing large GB issues to the market. Literature reports size of the issue as a key factor for bond investments [20,62,63]. Therefore, without large GB issues, investors may be discouraged from making GB investments.

If the issuer does not demonstrate reliable green credentials through their GB prospectus or issuing framework, investors must obtain a second opinion before making the investment. This creates a high transaction cost for investors and restricts investment decisions. When explaining the reasons for the high cost associated with second-party reviews, [16] focused their attention on three key factors. First, because there is no standardised methodology for the majority of second-opinion providers, reviews can vary, even within the same provider. This causes investors to obtain separate reviews on green credentials for each GB issue, even if the issues are made by the same issuer. Second, the opinion can be biased. Most second-party opinion providers are involved in the preparation of GB frameworks by issuers and the review of the same for investors. Third, reviews generally do not provide disclosures on the environmental impact of the bond. Moreover, reviewers do not monitor the use of proceeds throughout the bond term and do not undertake the liability for the project’s validation.

The [39] report in 2016 discussed the problems in the GB market in their study report on the potential of GB finance for resource-efficient investments. According to the study, five major barriers limit the growth of the GB market, especially in countries that have not yet actively participated in the GB market. These barriers include the lack of: (1) GBs and green project pipelines, (2) aggregation mechanisms for green projects, (3) a GB definition and framework, (4) information and market knowledge, and (5) clear risk profiles of green investments.

The analysis indicates that credibility concerns represent a recurring theme across documents and stakeholder interviews; however, the intensity and manifestation of this concern may vary across jurisdictions. The low market credibility is due to the high-cost requirement for GB issuers to confirm green credentials and to monitor them until bond maturity. Accordingly, the absence of appropriate definitions and standards can be identified as a common problem for both investors and issuers to make GB investments at low risk and low cost. Furthermore, findings revealed that the barriers identified in 2013 still appear as obstacles to market growth. Although different institutions and governments are making policy guidelines to overcome these issues, they have yet to be accepted by market participants.

The barriers that hinder GB market development were also discussed with all interviewees. The lack of appropriate GB supply and demand, lower credit ratings, lack of awareness of GBs as a source of financing, and high administrative and issuance costs were identified through the document review as the main problems that limit the growth of the GB market. These findings were further confirmed by participants during the interviews.

When discussing the challenges in the market, the first two respondents agreed regarding the unavailability of investment opportunities at the right time, overpricing, high administration costs and market illiquidity:

*It’s just the whole process of finding the right bonds at the right price, with the right criteria from the green perspectives, at the right time. So just having all those things coming to place at once.*

(Respondent 1)

*The over pricing could possibly be an issue … if it’s not consistent with the mainstream market and that would be adding cents for us, and the other issue is that, if you have distributing players who trying to do greenwash, that would be problematic, but hopefully we would get over that by making aware with our connecting counterparties and do our own due diligence. But that could be a problem.*

(Respondent 1)

*If you are an issuer of debt to do Green Bonds, it’s much higher reporting requirements by the clients, you got to do all that stuff, which is what you are asking for, but you don’t get any proceed on that. So, you know, we, as investors, are asking for people to do more work for no financial gain. Most of the ones that have issued to date pay by the current debt base by previously approved concept or just other systems to allow it or to diversify their investor base. I know what Queensland Treasury do, that was one of the reasons they were issuing Green Bonds. It’s just that they were talking to different investors and they got more investors in their books, which is better for them. So, in a way, issuers need another reason—not just to issue debt because they can issue debt in the mainstream market [and] they can get a lot of proceed in the same process without doing more works, as in Green Bond market. So that’s really the … their intention is probably the thing that I would say [is] limiting the growth in this market. People only do it if they can sell them potentially or to the best of buy investor base. It’s a good reason. But it’s not like if they issue Green Bonds, they save 10 basis points on the interest cost.*

(Respondent 1)

Respondent 2 supported and agreed with these opinions. Respondent 3, a representative of an issuer institution, also stressed the problem of burdensome administration associated with GB issues:

*Our main problem as a [institutional identification] issuing a GB has been to coordinate the required asset information across a range of diverse government agencies in order to develop our asset pool required for the GB. In addition, there are additional administrative requirements over and above a standard bond issuance that need to be maintained over the bond maturity. This has just taken extra time to date to bring the GB issuance to market. Twelve to 18 months, whereas a standard bond issuance can be arranged in less than seven days.*

(Respondent 3)

Additionally, the absence of tax incentives for GB investments was highlighted by Respondents 1 and 2, for example:

*As far as I know, there are tax incentives in other countries associated with the projects. And in Australia, like if you make a wind farm, there are tax incentives associated with the project itself, that not with the source of financing of that.*

(Respondent 2)

Respondents 4 and 5 identified the lack of awareness of GB benefits, existing international guidelines and standards, supply of labelled GBs and government intervention as barriers to scaling up the GB market. Additionally, Respondent 4 stated that the lack of information compared with current requirements, listings, reviewers and ratings is also a problem to overcome. This evidence concerning barriers in the GB market validated the findings of the document analysis.

### Theme # 04: Recommendations to overcome underlying challenges in the Green Bond market

The literature has made several suggestions to scale up the GB market to meet global requirements by overcoming the identified obstacles [Ex: 11,34,55].

The recommendations suggested by [16] include: promotion of the integrity of GBs and raise international awareness of their benefits, providing technical assistance for developing local GB guidelines, providing technical assistance for local currency bond market development, reducing costs of GB issuance and reporting, developing GB indices, ratings and stock exchange lists, labelling qualified GBs, promoting international collaboration to facilitate cross-border green capital flows, incubate local green investors, and enhancing the role of MDBs/development financial institutions (DFIs) and public entities when developing GB. They emphasised that the implementation of these suggestions should be decided by each country at the national level by considering the market size and country-specific requirements.

Furthermore, the *European Union* [39] suggested that governments should play a key role in the GB market to up scale the GB market. The report recommended mechanisms for public sector involvement, along with its costs and benefits. Table 3 illustrates the mechanisms suggested by the [39] based on the report of Adelphi/COWI (2016) for reducing GB market bottlenecks (Table 4).

**Table 4:** Summary of government-sector measures for overcoming GB market barriers.

Measures	Cost and benefits
**Demand-side measures**	
Public investment in GBs	Administrative costs are low to moderate; actual cost of implementation can be significant; potentially high influence if there is a lack of demand from private actors
**Supply-side measures**	
Public issuance of GBs	Administrative costs vary depending on familiarity of institutions with the issuance of bonds; the measure can have a significant leveraging effect
Public-sector support for aggregation and securitisation	Administrative costs are low to moderate; transaction costs can be substantial; the influence of the measure is potentially very large
Credit enhancement by public financing institutions	Administrative costs and actual costs of this measure are moderate to high; the influence of the measures is moderate
**Supply- and demand-side measures**	
Public facilitation of cooperation between GB market actors	Administrative costs are low; influence of the measure is potentially high
Tax incentives for GBs	Administrative costs are moderate; actual cost can be considerable; influence of tax incentives varies depending on the scope and extent of the tax
Support for standardisation of GB definition and framework	Administrative costs are moderate; influence of the measure could be very high
Preferential treatment of GBs in monetary regulation central bank strategy	Administrative costs are moderate; influence of the measure could be very high

Source: European Union (2016).

Regulators in the GB market expressed that it is timely and important to simplify the GB issuance framework, while maintaining its quality:

*Regional regulators should take a broad temperature of what global investors want and provide robust structures so that the domestic or regional issuers can have good access to the international market while maintaining the integrity of the bonds.* (Mark Robinson, Manager – Sustainability Services at DNV GL Business Assurance – ASIFMA Conference, 2018 as in [3])

In support of the above statement, at the same conference, Sean Kidney stated that there is an urgent need to simplify GB requirements in a manner that the frameworks and guidelines are interpreted with good judgment. He explained that simplifying frameworks without lowering their quality would help bring the GB market to the required scale:

*We need to grow markets in the areas that we need to achieve emission savings fast. We need to get scale quickly … We need to take away the work from the issuers because every time we have to explain to a treasurer one more thing they have to do, it is one other reason why they do not do it.* (Sean Kidney – ASIFMA Conference, 2018 as in [3]).

Participants provided opinions about the recommendations identified during the document analysis. They all highly recommended government involvement in the GB market as the main solution to overcoming the challenges. Improved impact reporting, strong external reviews and ratings were also recommended by Respondent 4:

*Smarter impact reporting is needed (not necessarily more information, but the right information in clear communication to investors). From an environmental perspective, improved impact reporting would help provide more clear benefit … I think green integrity can be maintained through stronger external reviews and ratings.*

(Respondent 4)

Moreover, participants expressed views on the additional features to be considered by GBPs to make the market more attractive. All agreed with the importance of external reviews and second-party opinions, while Respondents 1 and 2 stressed the need to add a human rights risk assessment segment to the existing GBPs:

*I think they should also include a human rights risk assessment segment. Because … yes, they can actually assess the greenness of the bond, but you need to be respectful to the labour standards of the people working with the projects during the period and also the local communities are there. So, I think there should be a human rights aspect as well.*

(Respondent 1)

After considering the interview findings, we were satisfied with the validity of the document analysis. Overall, the findings indicated that the upscaling of the GB market is essential in order to assist in meeting the climate change challenges around the world. GBPs and the CBS can play a significant role in this process by improving existing guidelines and criteria in line with market requirements. In addition, government agencies should intervene in the market to enhance market credibility and reduce default risk.

Importantly, the principal contribution of this study lies not merely in identifying barriers within the GB market, but in integrating stakeholder-level evidence with authoritative global policy documents. While institutional reports often present normative recommendations and theoretical frameworks, our interview findings provide contextual support for how these frameworks operate in practice, exposing administrative burdens, pricing tensions, definitional ambiguities, and credibility concerns faced by market actors. By synthesizing documentary discourse with practitioner insight, this study bridges the gap between theoretical policy design and practical implementation challenges, thereby offering a grounded understanding of why scaling the GB market remains complex despite extensive regulatory development.

## 5. Discussion and conclusion

This study was motivated by several issues identified in the GB market. First, the GB market development was identified as essential as a new source of financing for ‘green’ projects. This was evident by the CBI’s CEO statement that GBs are ‘the Trojan horse’ of sustainable financing. However, the market is not at the scale needed to meet worldwide green finances. Different stakeholders characterise the GB market. Therefore, identifying the ‘greenness’ of a project financed by GBs is problematic. The low credibility of the GB issuance process is highly questioned by potential investors, who express concern regarding the possibility of greenwashing. Therefore, an in-depth study of the GB market is timely to identify the reasons for the limitations in this market. Second, while prior studies have substantially advanced understanding of Green Bond pricing, investor behaviour, and market performance, relatively limited attention has been given to integrating global policy frameworks with stakeholder perceptions into a unified conceptual explanation of barriers to scaling the Green Bond market. This study addresses that gap by synthesising documentary evidence with exploratory stakeholder insights. A better understanding of the market will help researchers build new theories of the GB market and suggest improved practices and recommendations.

Accordingly, we used a qualitative research approach to identify the issues associated with GBs, the factors hindering market development, investors’ expectations of the market and the recommendations provided by market experts and other stakeholders to minimise current problems. Document analysis was conducted to analyse publications issued by market leaders and regulators. The identified factors were further validated through opinions provided by stakeholders in the market. Five executives who represented investor, issuer and external reviewer institutions were contacted through interviews for data validity purposes. The appropriateness of the process of data collection, employment of methodologies and data analysis was confirmed through peer debriefing.

The findings of this study revealed a few key factors about the GB market. First, despite having different assessment criteria for the ‘greenness’ of projects, these criteria are not quantified or standardised. Second, both the demand and supply sides of the GB market do not meet the required level. Moreover, the high administration cost, low knowledge about the market, low standardisation and difficulty obtaining credit ratings limit GB issues. Meanwhile, the absence of large-scale issues, low market liquidity and fear for greenwashing because of low market credibility limit GB investments. As a result of the fear of greenwashing, investors require highly transparent disclosures from issuers before and after issuing GBs. However, the high cost associated with reporting requirements discourage issuers from ongoing reporting. Third, investors consider both quantitative and qualitative factors before making green investment decisions. The main financial factors are the risk–return profile of the particular bond, issuance currency, credit rating, size of the bond issue, bond maturity and bond pricing. Other considerations include ESG factors, the issuer’s reputation for eco-friendly business, the issuer’s other projects, and government intervention. We combine these findings into a theoretical model as presented below in Fig 1.

**Fig 1:**
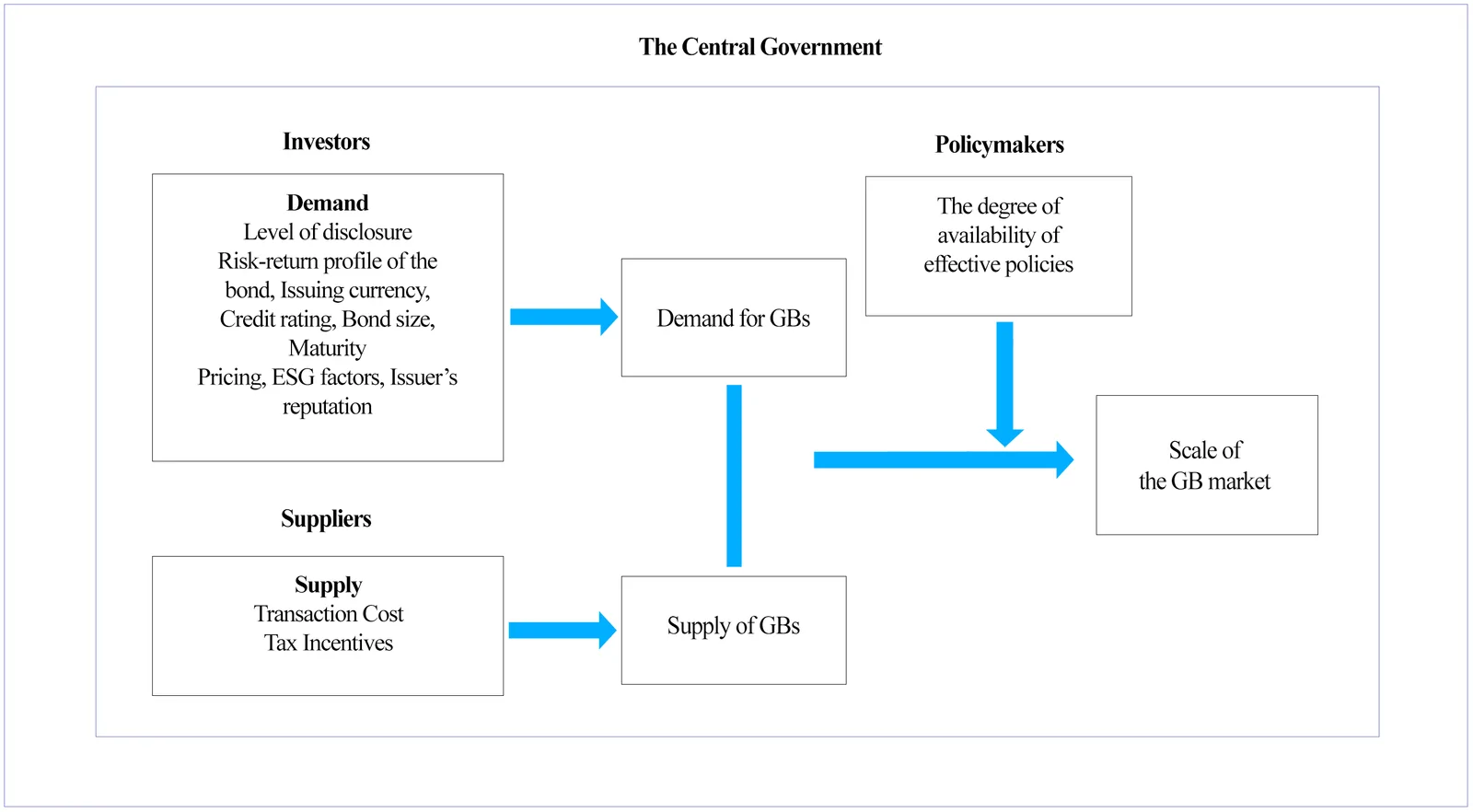
Theoretical framework developed based on the findings: Stakeholder perception and scaling up the green bond market through policy recommendations.

Fig 1 presents the theoretical framework developed inductively from the study’s findings. The model integrates insights from stakeholder interviews and global documentary analysis to illustrate how demand-side factors (e.g., disclosure quality, risk–return profile, pricing, ESG considerations, and issuer reputation) and supply-side constraints (e.g., transaction costs, administrative burden, and tax incentives) jointly influence the scale of the Green Bond market.

The integrated framework demonstrates that the challenges of definitional ambiguity can be interpreted from three complementary theoretical perspectives. Stakeholder theory explains how divergent expectations among issuers, investors and regulators create competing interpretations of “greenness.” Institutional theory highlights how fragmented regulatory systems and evolving norms prevent convergence towards common market standards. Market failure theory explains how information asymmetry and negative externalities reduce investor confidence and weaken incentives for market participation. Together, these perspectives provide a richer explanation of why persistent structural barriers continue to constrain the scaling of the GB market beyond what any individual theory can explain independently. Further, the model demonstrates that scaling the Green Bond market depends not solely on financial performance, but on coordinated stakeholder alignment supported by effective policy mechanisms.

Considering the study’s findings, several important recommendations can be made to overcome the barriers to scaling up the global Green Bond (GB) market. Interview participants consistently highlighted definitional inconsistency as a structural constraint. Document analysis similarly revealed regulatory fragmentation across the GBPs, CBS, and national taxonomies. These findings suggest that greater cross-jurisdictional harmonization of GB standards may reduce interpretive ambiguity and enhance investor confidence. However, full harmonization may be politically and economically constrained, particularly given differing national transition priorities. Therefore, incremental alignment through mutual recognition frameworks may represent a more feasible pathway.

Participants also emphasized reporting burdens and credibility concerns. This aligns with documentary evidence highlighting greenwashing risks and inconsistent impact reporting. Strengthening post-issuance disclosure requirements and third-party verification mechanisms could improve credibility and reduce perceived greenwashing risks. However, increased reporting requirements may disproportionately burden smaller issuers, suggesting that tiered compliance mechanisms could improve inclusivity.

Moreover, several respondents suggested that government backing could catalyse market growth. Targeted fiscal incentives, credit guarantees, or public co-financing mechanisms may reduce entry barriers and stimulate supply. The effectiveness of such measures is likely context-dependent, particularly in emerging markets with limited fiscal space.

It is important to recognize that the feasibility of these measures varies across institutional contexts. Developed markets with mature regulatory infrastructures may be better positioned to implement harmonized taxonomies and enhanced disclosure regimes, whereas emerging markets may prioritize capacity-building and phased implementations. Accordingly, the proposed policy pathways should be interpreted as adaptive frameworks rather than universal prescriptions.

Our study contributes to both the finance and sustainable development literature and practice. While prior empirical studies have examined pricing dynamics, environmental impacts, and governance challenges in the GB market, fewer studies have qualitatively synthesized global policy discourse with stakeholder-level insights to explore implementation barriers. Further, this study comprehensively reports a number of issues regarding the GB market’s definitions, barriers, expectations and recommendations, thereby providing stakeholders a better understanding about the GB market. We identified vital areas to be streamlined to upscale the GB market. By doing so, this study contributes to overcoming a pressing global issue—financing projects and new technologies to mitigate and adapt to climate change problems worldwide.

By combining global documentary analysis with stakeholder interviews, our study contributes an integrative perspective that connects macro-level regulatory discourse with micro-level market realities. This dual approach advances understanding beyond purely empirical pricing studies or normative policy reports, positioning the GB market as an evolving institutional field shaped by regulatory ambiguity, market incentives, and stakeholder perceptions. In doing so, the research provides a structured foundation for future empirical testing and comparative international studies.

### Limitations and scope

Our study is based on qualitative analysis of selected institutional documents and five expert interviews, primarily representing developed-market perspectives. While triangulation enhances internal validity, the findings should not be interpreted as statistically generalizable. Instead, they provide analytically grounded insights into structural barriers and stakeholder perceptions within the GB market. Future research may employ large-scale surveys or cross-country qualitative designs to further validate and extend these findings. Furthermore, another key limitation concerns the geographically concentrated interview sample. Although documentary evidence reflects global developments, stakeholder interviews were conducted primarily within the Australian and European markets. Future research should extend qualitative inquiry to emerging markets and diverse institutional contexts to enhance comparative generalizability. Given the rapid evolution of sustainable finance frameworks after 2021, including the expansion of green taxonomies and post-pandemic recovery instruments, stakeholder perceptions may have evolved. Longitudinal qualitative research would provide valuable insights into how these perceptions have shifted over time.

## Supporting information

S1 File This is the Coding Framework. (PDF)
