# Supplementary material for: Scaling up the global Green Bond market: Integrating global policy frameworks with stakeholder perceptions from Australia and the United Kingdom
Source: PLoS One. 2026 Oct 5;21(10):e0338998. doi: 10.1371/journal.pone.0338998 (PMC13637898; doi:10.1371/journal.pone.0338998)
Supplement: Supplementary file 1 [file pone.0338998.s001.pdf]

Supplementary Information

Appendix 01: Qualitative Codebook and Coding Framework

Purpose of this appendix

This appendix provides the complete qualitative coding framework used in the study to enhance transparency and reproducibility. The analysis followed the three-stage coding procedure proposed by Strauss and Corbin (1998), consisting of open coding, axial coding, and selective coding. Documentary evidence served as the primary analytical source, while stakeholder interviews were used to validate and contextualize the themes emerging from the documentary analysis.

The coding process was iterative, with continuous comparison between documentary evidence and interview data until conceptual consistency was achieved.

Table S1: Coding Framework

First-order empirical code	Second-order analytical category	Aggregate theme	Evidence source
Lack of common green definition	Definitional inconsistency	Theme 1 – Definition of Greenness	ICMA, OECD, IISD
Multiple interpretations of "green"	Definitional inconsistency	Theme 1	ICMA, World Bank
Country-specific green definitions	Regulatory fragmentation	Theme 1	PBoC, SEBI
Green taxonomies differ across jurisdictions	Regulatory fragmentation	Theme 1	EU Taxonomy, China Taxonomy
Lack of universally accepted assessment criteria	Assessment uncertainty	Theme 1	CBI
Greenwashing concerns	Credibility concerns	Theme 1	OECD, IISD
Independent certification required	External assurance	Theme 1	Respondent 3
Investors perform own due diligence	Verification behaviour	Theme 2 – Investor expectations	Respondent 1
Transparent disclosure required	Disclosure expectations	Theme 2	World Bank Symposium
Annual impact reporting	Reporting expectations	Theme 2	INCR
Third-party verification	Assurance expectations	Theme 2	Respondent 4
Issuer reputation	Issuer credibility	Theme 2	Respondents 1 & 2
Financial return expectations	Investment characteristics	Theme 2	World Bank

Pricing comparable to conventional bonds	Pricing expectations	Theme 2	Respondent 3
Currency risk	Investment considerations	Theme 2	Respondent 2
Government backing	Credit assurance	Theme 2	Respondent 2
ESG performance	Sustainability expectations	Theme 2	Respondent 1
High certification costs	Administrative burden	Theme 3 – Market Barriers	OECD
High reporting costs	Administrative burden	Theme 3	ASIFMA
High transaction costs	Cost barriers	Theme 3	OECD
Small issue size	Liquidity constraints	Theme 3	IISD
Low market liquidity	Liquidity constraints	Theme 3	OECD
Low credit ratings	Credit constraints	Theme 3	OECD
Limited bankable projects	Supply constraints	Theme 3	CBI
Limited investor awareness	Awareness constraints	Theme 3	GFSG
Limited issuer awareness	Capacity constraints	Theme 3	GFSG
Fragmented regulations	Institutional fragmentation	Theme 3	OECD
Inconsistent reporting standards	Reporting inconsistency	Theme 3	CBI
Lack of harmonization	Regulatory fragmentation	Theme 3	Respondent 5
Harmonized global standards	Standardization	Theme 4 – Policy Recommendations	OECD
Develop global taxonomy	Regulatory harmonization	Theme 4	EU
Improve disclosure requirements	Transparency enhancement	Theme 4	World Bank
Strengthen impact reporting	Reporting enhancement	Theme 4	INCR
Simplify reporting requirements	Administrative reforms	Theme 4	ASIFMA
Reduce certification costs	Cost reduction	Theme 4	OECD
Government incentives	Public policy	Theme 4	OECD
Tax incentives	Financial incentives	Theme 4	OECD
Capacity building	Market development	Theme 4	GFSG
Investor education	Capacity development	Theme 4	GFSG
International cooperation	Institutional coordination	Theme 4	Respondent 5

Table S2: Illustration of the Coding Process

Representative evidence	Open code	Axial code	Selective theme
"The Green Bond Principles provide the basis for defining green bonds."	GBPs as benchmark	Definitional consistency	Theme 1
"Country-specific standards create inconsistencies."	Regulatory differences	Regulatory fragmentation	Theme 1
"Investors conduct their own due diligence."	Investor verification	Verification behaviour	Theme 2
"Transparency is important before investment."	Disclosure	Disclosure expectations	Theme 2
"Pricing remained similar to conventional bonds."	No pricing advantage	Pricing expectations	Theme 2
"Reporting requirements increase issuing costs."	Reporting burden	Administrative burden	Theme 3
"Independent certification improves credibility."	External review	Credibility enhancement	Theme 3
"Government intervention is necessary."	Policy support	Policy intervention	Theme 4
"International harmonization is essential."	Harmonization	Standardization	Theme 4
"Green taxonomies should be aligned internationally."	Common taxonomy	Regulatory harmonization	Theme 4

Table S3: Relationship between documentary analysis and interviews

Documentary finding	Supporting interview evidence	Contribution of interview
Definitional inconsistency	Respondents 4 and 5 acknowledged reliance on GBPs while recognizing differences across jurisdictions.	Validated documentary findings
Transparency is essential	Respondent 1 emphasized issuer reporting and disclosure.	Contextual validation
Investors conduct independent due diligence	Respondents 1 and 2 explained internal investment screening procedures.	Practical illustration
Reporting requirements create burdens	Respondents 3 and 4 discussed increasing compliance costs.	Contextual support
Government intervention is needed	Respondent 2 highlighted the importance of sovereign and supranational issuers.	Validation
International harmonization	Respondent 5 emphasized gradual harmonization of standards.	Validation

Coding procedure

The qualitative analysis followed the grounded theory coding procedures proposed by Strauss and Corbin (1998). During open coding, documentary sources and interview transcripts were examined line by line to identify recurring concepts relevant to the research objectives. Initial codes were generated inductively from institutional reports, policy documents, industry publications and interview transcripts.

During axial coding, conceptually similar first-order codes were grouped into broader analytical categories, including regulatory fragmentation, disclosure expectations, administrative burden, liquidity constraints and policy interventions. Constant comparison was undertaken throughout this stage to ensure conceptual consistency across documentary and interview evidence.

Finally, selective coding integrated these analytical categories into four aggregate themes corresponding to the study objectives: (1) definition of greenness, (2) investor expectations, (3) barriers to scaling the Green Bond market, and (4) policy recommendations.

Documentary analysis constituted the primary analytical foundation, while interview evidence served to validate and contextualize the emerging themes rather than generate independent findings.

Analytical audit trail

To enhance methodological transparency, an audit trail was maintained throughout the coding process. Emerging codes identified through documentary analysis were continuously compared with evidence from stakeholder interviews to assess conceptual consistency. When interview participants provided additional contextual insights, the documentary sources were revisited to determine whether similar patterns existed. This iterative process ensured that the aggregate themes remained primarily grounded in documentary evidence while being strengthened by stakeholder validation.

Dependability and credibility

Several procedures were employed to enhance the trustworthiness of the qualitative analysis.

- Documentary analysis undertaken before the interviews.
- Interviews were used solely to validate and contextualize the documentary findings.
- Constant comparison was employed throughout coding.
- Coding decisions were documented to maintain consistency.
- The three-stage coding process (open, axial and selective coding) provided a transparent analytical structure.
- Representative quotations and documentary evidence were retained to preserve the link between the raw data and the final themes.
